# Rapid Determination of Oxygen Saturation and Vascularity for Cancer Detection

**DOI:** 10.1371/journal.pone.0082977

**Published:** 2013-12-16

**Authors:** Fangyao Hu, Karthik Vishwanath, Justin Lo, Alaattin Erkanli, Christine Mulvey, Walter T. Lee, Nimmi Ramanujam

**Affiliations:** 1 Department of Biomedical Engineering, Duke University, Durham, North Carolina, United States of America; 2 Department of Biostatistics and Bioinformatics, Duke University, Durham, North Carolina, United States of America; 3 Division of Otolaryngology–Head and Neck Surgery, Duke University Medical Center, Durham, North Carolina, United States of America; 4 Section of Otolaryngology–Head and Neck Surgery, Veterans Administration Medical Center, Durham, North Carolina, United States of America; Glasgow University, United Kingdom

## Abstract

A rapid heuristic ratiometric analysis for estimating tissue hemoglobin concentration and oxygen saturation from measured tissue diffuse reflectance spectra is presented. The analysis was validated in tissue-mimicking phantoms and applied to clinical measurements in head and neck, cervical and breast tissues. The analysis works in two steps. First, a linear equation that translates the ratio of the diffuse reflectance at 584 nm and 545 nm to estimate the tissue hemoglobin concentration using a Monte Carlo-based lookup table was developed. This equation is independent of tissue scattering and oxygen saturation. Second, the oxygen saturation was estimated using non-linear logistic equations that translate the ratio of the diffuse reflectance spectra at 539 nm to 545 nm into the tissue oxygen saturation. Correlations coefficients of 0.89 (0.86), 0.77 (0.71) and 0.69 (0.43) were obtained for the tissue hemoglobin concentration (oxygen saturation) values extracted using the full spectral Monte Carlo and the ratiometric analysis, for clinical measurements in head and neck, breast and cervical tissues, respectively. The ratiometric analysis was more than 4000 times faster than the inverse Monte Carlo analysis for estimating tissue hemoglobin concentration and oxygen saturation in simulated phantom experiments. In addition, the discriminatory power of the two analyses was similar. These results show the potential of such empirical tools to rapidly estimate tissue hemoglobin in real-time spectral imaging applications.

## Introduction

Numerous studies have shown that the early detection and treatment of oral and cervical cancers significantly improve survival rates [1]–[8]. Detection of precancerous and cancerous oral lesions is mostly accomplished through visual inspection followed by the biopsy of suspicious tissue sites. For cervical cancer screening, the Papanicolau test or Pap smear is the standard of care. If the Pap smear is positive, colposcopy (visualization of the acetic acid stained cervix with a low power microscope) and biopsy are performed. An effective cancer screening and diagnostic program often requires both sophisticated and expensive medical facilities with well-trained and experienced medical staff. In developing countries, however, there is the absence of appropriate medical infrastructure and resources to support the organized screening and diagnostic programs that are available in the U.S. Therefore, there is a critical global need for a portable, easy-to-use, reliable and low cost device that can rapidly screen for oral and cervical cancer in low-resource settings.

UV-visible (UV-VIS) diffuse reflectance spectroscopy, which can be used to measure tissue absorption and scattering, has shown potential for the early diagnosis of cancers in the cervix and oral cavity [9]–[24]. The absorption and scattering coefficients of epithelial tissues reflect the underlying physiological and morphological properties [25]. In the UV-VIS band, the dominant absorbers in oral and cervical tissues are oxygenated and deoxygenated hemoglobin, arising from blood vessels in the stroma. Light scattering is primarily associated with cell nuclei and organelles in the epithelium, as well as collagen fibers and cross-links in the stroma. Neoplastic tissues exhibit significant changes in their physiological and morphological characteristics that can be quantified optically. The contribution of absorption in the stromal layer is expected to increase with neovascularization and angiogenesis, and the oxygen saturation in blood vessels is expected to decrease as the neoplastic tissue outgrows its blood supply. Stromal scattering is expected to decrease with neoplastic progression due to degradation of extracellular collagen networks. [11], [25]–[29]. However, epithelial scattering is expected to increase due to increased nuclear size, increased DNA content, and hyperchromasia [25]–[27], [30]. UV-VIS diffuse reflectance spectroscopy has a penetration depth that can be tuned to be comparable to the thickness of the epithelial layer or deeper to probe both the epithelial and stromal layers [17], [25], [31].

Our group has developed a UV-VIS diffuse reflectance spectroscopy system with a probe geometry that is most sensitive to changes in the stroma and a scalable inverse Monte Carlo (MC) reflectance model to rapidly measure and quantify tissue optical properties [32], [33]. Chang et al. [10] used the spectroscopic system and the MC model to identify optical biomarkers that vary with different grades of cervical intraepithelial neoplasia (CIN) from normal cervical tissues in 38 patients. Total hemoglobin was found to be statistically higher in high-grade dysplasia compared with normal and low grade dysplasia (P<0.002), whereas scattering was significantly reduced in dysplasia compared with normal tissues (P<0.002). Beumer et al. used the same UV-VIS diffuse reflectance spectroscopy system in an *in vivo* clinical study in which 21 patients with mucosal squamous cell carcinoma of the head and neck were evaluated [34]. All 21 patients underwent panendoscopy and biopsies were taken from the malignant and the contralateral normal tissues. Diffuse reflectance spectra were measured prior to biopsy. The vascular oxygen saturation (SO_2_) was found to be statistically higher in malignant tissues compared to non-malignant tissues (P = 0.001).

The most efficient and effective strategy for the prevention of advanced cervical or oral cancers in resource-limited settings is to see and treat the patient in a single-visit, thus obviating the need for a multi-tiered system such as that in the U.S. where screening, diagnosis, and treatment entail three or more visits to the healthcare facility. For example, guidelines have been written by the Alliance for the Prevention of Cervical Cancer (APCC) on strategies for screening cervical cancer in resource-limited settings [35]. Their recommendation is visual inspection with acetic acid (VIA), followed by treatment of the pre-cancerous lesions using cryotherapy (freezing) [36]–[38], which can be carried out by physicians, nurses or midwives. An effective screening/diagnostic strategy that can allow for immediate treatment intervention needs to be able to survey the entire region of interest. Further, the detection strategy should be minimally affected by operator bias or subjective interpretation of images collected from the region of interest. Our current system enables quantitative determination of tissue physiological endpoints, but is limited to evaluating localized regions of the tissue. To survey the entire field of view, it is important to scale the single-pixel fiber-based system into an imaging platform and develop algorithms that can quantify these spectral images. However, development of simple imaging systems requires a significant consolidation of the number of wavelengths, so that imaging spectrographs and broad-band thermal sources can be replaced by simple cameras and LEDs.

The goal of this study was to demonstrate a simple ratiometric analysis for the quantitation of tissue SO_2_ and total hemoglobin concentration ([THb]) using a small number of wavelengths in the visible spectral range as a strategy for implementation of rapid surveillance of pre-cancers and cancers in a screening population in resource-limited settings. Several previously published studies have utilized simple ratiometric analyses to compute [THb] or SO_2_ from reflectance spectra. For example, ratiometric analyses have been developed to extract SO_2_ using ratios at two wavelengths, one where the local differences between the extinction coefficients of oxy- and deoxy- hemoglobin are maximal, and one isosbestic wavelength, where the extinction coefficients of oxy- and deoxy- hemoglobin are the same. In one study [39], the ratio of 431/420 was computed and used to calculate SO_2_. However, this study did not account for the effects of tissue scattering. Another study [40] used the optical densities at two isosbestic points, 520 and 546 nm, to determine the contribution of scattering and use the optical density at 555 and 546 nm to extract SO_2_ through a linear equation. However, this study did not explore the impact of changes in [THb] on the ratios. Our group has previously developed a ratiometric analysis [41] which computes reflectance ratios at the isosbestic wavelengths of hemoglobin, and this analysis was able to rapidly calculate [THb] independent of tissue scattering and SO_2_. For this particular ratiometric analysis, the ratio of the intensities at one visible wavelength (452, 500, or 529 nm) to one ultraviolet wavelength (390 nm) from a diffuse reflectance spectrum was used to extract [THb] using a linear analytical equation. However, this analysis would require an ultraviolet source, which is relatively expensive compared to ubiquitous visible wavelength light sources. In this manuscript, we describe a simple and analytical ratiometric analysis to extract both [THb] and SO_2_ in the visible wavelength range that addresses the limitations of previous work by our own group and others. It utilizes two or more intensities at different wavelengths from a diffuse reflectance spectrum and calculates appropriate ratios from them. The derived ratios are then converted to [THb] or SO_2_ using analytical equations. Our proposed analysis utilizes only three wavelengths (539, 545 and 584 nm), all in the visible part of the spectrum where light emitting diodes (LEDs) are readily available. We also tested our ratiometric analysis with full spectral MC simulations and experimental phantoms to ensure minimal sensitivity to scattering. In addition, our ratiometric analysis also accounts for [THb] when computing SO_2_.

## Methods

Wavelengths were chosen from 500 nm to 600 nm (visible spectrum) in order to leverage relatively low priced light sources such as LEDs. In addition, deoxy- and oxy-hemoglobin have distinct absorption features in the visible spectrum. Five isosbestic wavelengths and five other wavelengths where the difference of extinction coefficients between deoxy- and oxy-hemoglobin are largest were used to calculate [THb] and SO_2_, respectively. Table 1 lists these wavelengths, which provide a total of ten possible combinations (pairs of isosbestic wavelengths), at which ratios were tested for extraction of [THb] and 25 wavelength combinations at which the reflectance ratios were tested (one isosbestic and one maximal-difference wavelength) for extraction of SO_2_.

**Table 1:** Wavelengths used for extraction of [THb] and SO_2_.

Isosbestic Wavelengths for [THb] (nm)	Wavelengths for Oxygen Saturation (SO_2_) (nm)
500	516
529	539
545	560
570	577
584	593

**Figure 1:**
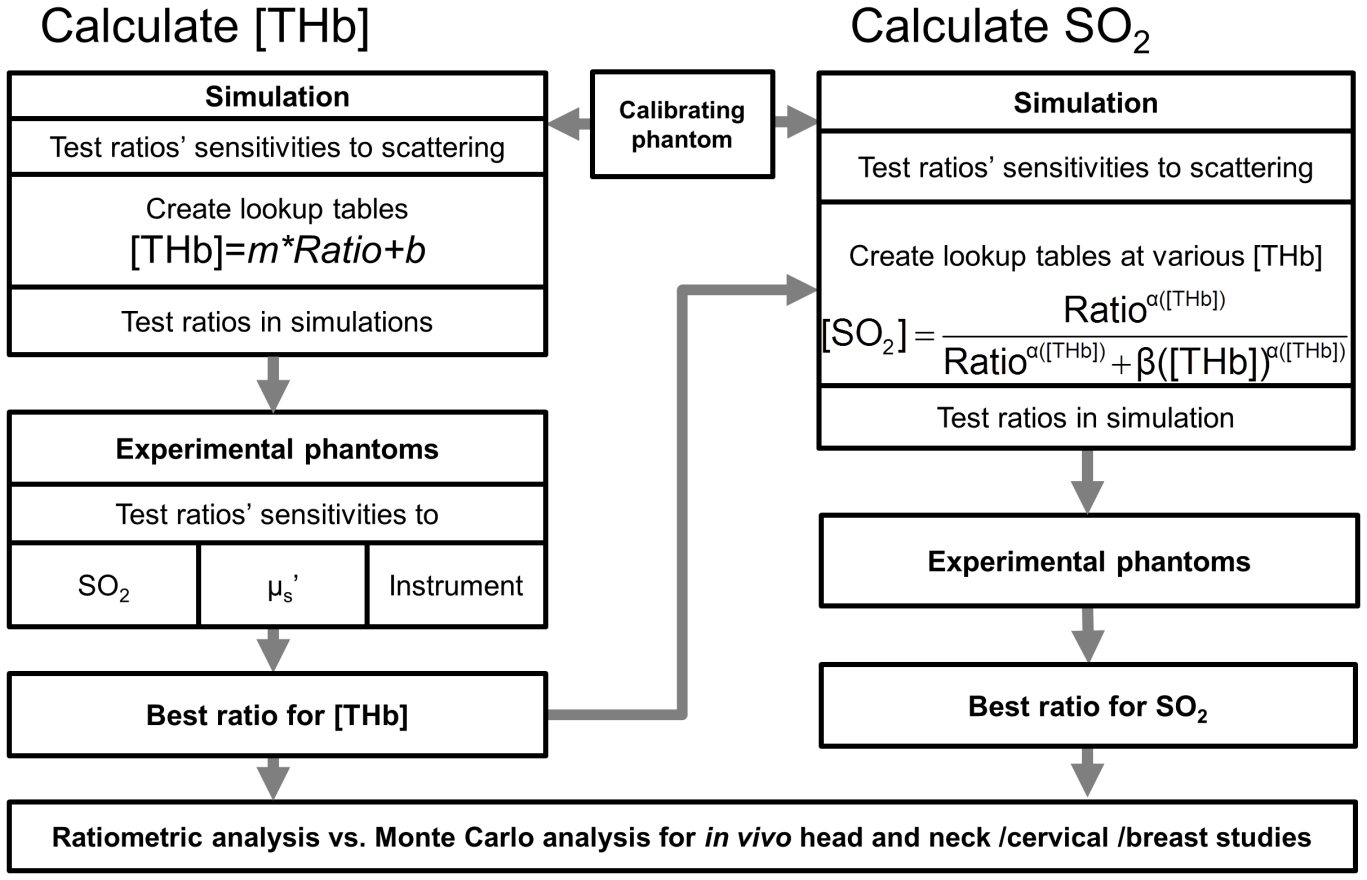
Flow chart illustrating the ratiometric analysis for [THb] and SO_2_ estimation.

Figure 1 briefly provides an overview of the ratiometric analysis including the steps involved in the selection of the best ratios for [THb] and SO_2_. Extractions of [THb] and SO_2_ were achieved in two steps. First, the reflectance ratio comprised of isosbestic wavelengths was used to extract [THb]. This was achieved by converting the reflectance ratio into [THb] using a linear equation. For each ratio at isosbestic wavelengths, independent sets of the coefficients *m* and *b* were generated using MC simulations. Next, the reflectance ratio at one isosbestic wavelength and one maximal-difference wavelength was converted into an SO_2_ value using a non-linear equation using the *α* (THb) and β (THb) coefficients. These coefficients were generated using MC simulations for each of the 25-reflectance ratios at every simulated [THb]. The extracted [THb] from the first step was used to select the appropriate non-linear logistic equation to convert the ratio of the isosbestic to maximum difference wavelength into the SO_2_ value. After the equations for [THb] and SO_2_ were developed, the ratiometric analysis was validated with experimental tissue mimicking phantoms. To show the clinical utility of this analysis and its independence to changes in instrumentation, the extractions using the selected ratios were then compared with those using the full spectral MC analysis in three different clinical studies carried out with different optical systems.

### Generating analytical lookup tables for [THb] and SO_2_ from reflectance ratios

Analytical equations to convert appropriate ratios into [THb] and SO_2_ values were determined using full spectral MC simulations. The forward full spectral MC model [42] was used to generate 24805 unique diffuse reflectance spectra. These reflectance spectra served as the simulated master set. Diffuse reflectance spectra were simulated by calculating the absorption and scattering spectrum between 350–600 nm. The absorption coefficients were calculated with the assumption that oxy- and deoxy-hemoglobin are the dominant absorbers in tissue. The sum of these two absorber concentrations gave the resulting [THb], which was varied between 5 and 50 µM in increments of 0.1 µM in the master set. The concentration of each hemoglobin species was varied to span the range of SO_2_ values from 0 to 1, in steps of 0.1. The reduced scattering coefficients, μ_s_', across the spectral range were determined using Mie theory for 1 µm polystyrene microspheres. Five different scattering levels were generated by increasing the number density of sphere concentrations. The wavelength-averaged (between 350∼600 nm) mean reduced scattering coefficients for these five scattering levels were 8.9, 13.3, 17.8, 22.2, and 26.6 cm^−1^. The resulting master set consisted of 24805 reflectance spectra, which represent the combination of all possible [THb] levels, with all SO_2_ levels, and all scattering levels (451×11×5 = 24805). These optical properties are similar to those used in our previous study [41]. The simulated reflectance spectra for the master set were created for a fixed fiber-probe geometry, as described previously [42]. Finally, an experimentally measured diffuse reflectance spectrum with the same fiber-geometry was used as a “reference” to calibrate the scale of the simulated spectra to be comparable to that of measured spectra.

To study the impact on extraction accuracy of the ratiometric analysis with increasing spectral bandpasses, we simulated additional bandpasses in the master set. The reflectance spectra were simulated for three different bandpasses (2 nm, 3.5 nm and 10 nm full width-half-maximum (FWHM) bandwidths) and resulted in 3 modified master diffuse reflectance sets (each containing 24,805 spectra). This was done by assuming each wavelength had a certain Gaussian bandpass of specified FWHM. Specifically, the reflectance at each wavelength in the simulated spectrum was convolved with a Gaussian distribution function with the specific bandpass. Equations to convert reflectance ratios into [THb] and SO_2_ were then generated separately for each of the three bandpass-modified master diffuse reflectance spectral sets.

**Figure 2:**
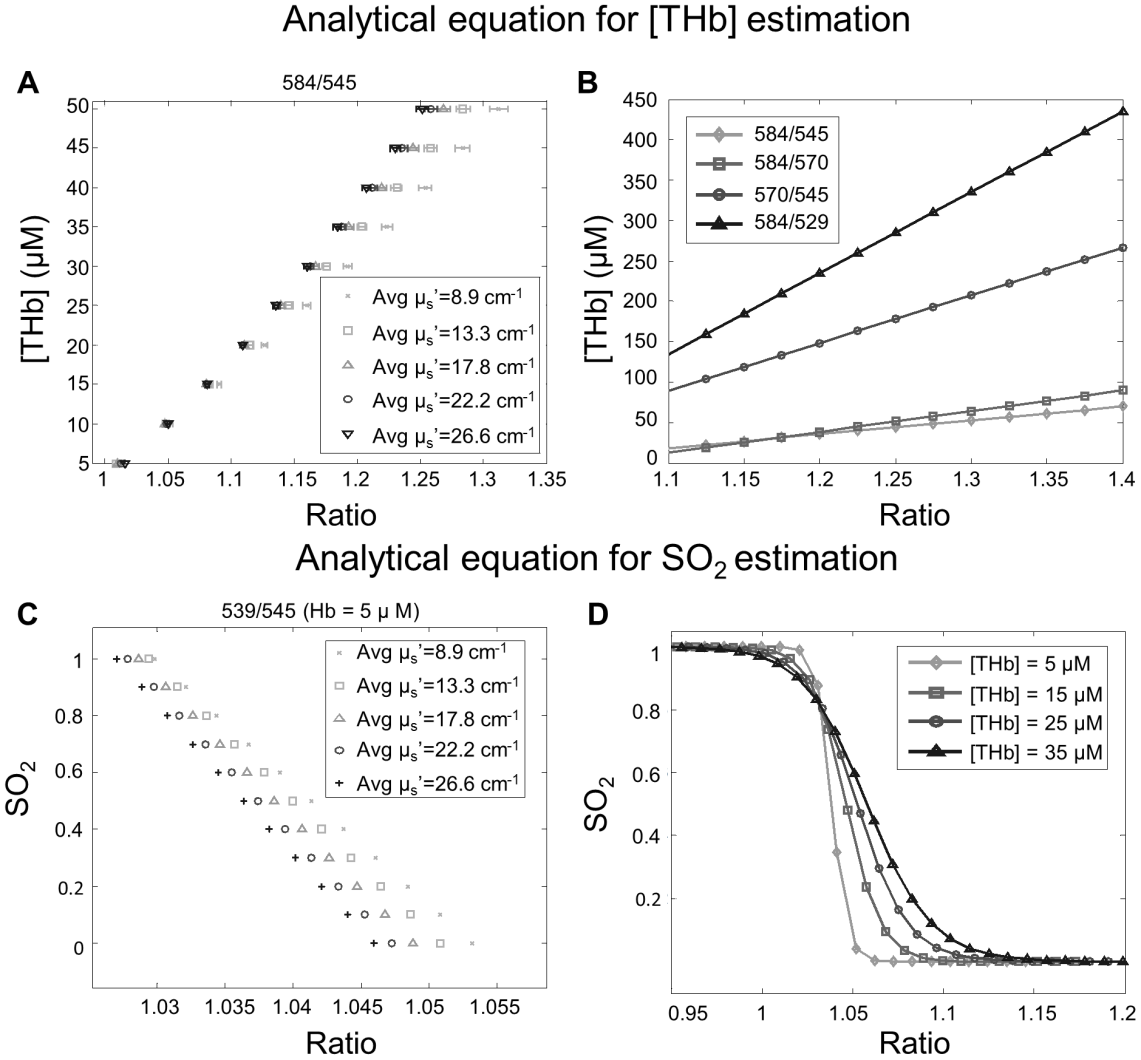
Steps for calculating the analytical equations. Steps for calculating the analytical equations: (A) Generating reflectance with various optical properties using forward analysis and derived Hb ratios. The horizontal error bars show the standard deviation of the ratios at SO_2_ levels from 0 to 1. The spreads are small because the ratios are derived from isosbestic points. (B) Example linear analytical equations of 584/545, 584/570, 570/545, and 584/529 for [THb] estimation. (C) Calculating SO_2_ ratios with several scattering levels at one [THb] (D) Hill curve equations were generated at several [THb] for each SO_2_ ratio. Only 539/545 is shown.

Figure 2 describes the development of analytical equations used to compute [THb] and SO_2_. A [THb] ratio, 584/545, and an SO_2_ ratio, 539/545, are shown as examples. For [THb] extraction, the reflectance ratio at a given wavelength-pair was computed from every simulated reflectance spectrum that had a fixed [THb]. Thus, there were 55 values for a given [THb] wavelength-ratio (across the 5 scattering levels and 11 SO_2_ levels). Eleven of these values were averaged across SO_2_, for each scattering level. For each of the ten isosbestic wavelength-pairs, the dependence of the reflectance ratio on [THb] was plotted across all SO_2_ levels and each scattering level, as shown in Figure 2A. Although the analysis consisted of 5–50 µM [THb] in steps of 0.1 µM, only 10 of the 451 [THb] levels are shown in the figure for easier interpretation of the data points. We evaluated the dependence of the reflectance ratio for a given wavelength-pair on tissue SO_2_ and scattering. The horizontal error bars at each scattering level show the spread of the reflectance ratio due to varying SO_2_ levels from 0 to 1. This reflects the sensitivity of the ratio to changes in SO_2_. The spread in the different symbols at each [THb] reflects the sensitivity of the ratio to scattering. The reflectance ratios at each [THb] were averaged across the 5 scattering levels and the 11 SO_2_ levels, and a linear analytical equation was generated for the averaged ratios. Figure 2B shows the linear analytical equations for 584/545, 584/570, 570/545, and 584/529 as examples.

In order to convert the reflectance ratio computed at a given SO_2_ wavelength-pair into an SO_2_ value, a non-linear logistic (Hill curve) equation was used. A unique Hill equation was generated for each of the 451 [THb] (5–50 µM in 0.1 increment steps) in the modified master set. The reflectance ratio for a given SO_2_ wavelength-pair, at a given [THb], was averaged across the five scattering levels (Figure 2C). This resulted in 11 averaged ratios for each SO_2_ wavelength pair, at each [THb]. The Hill coefficients were generated by fitting the 11 averaged ratios to the logistic equation. Since a total of 451 different [THb] values were used in the simulations, 451 different equations were generated for each SO_2_ wavelength-pair. Figure 2D shows the example figures of the Hill curves generated from the averaged ratios at different [THb] for 539/545.

### Determination of the best ratios from simulation and experimental phantoms

A total of 8 sets of reflectance spectra were used to validate the ratiometric analysis. The optical properties and collection parameters for these 8 phantom sets are summarized in Table 2. Phantom sets 1–3 were simulated with the scalable MC model, as described above. Phantom sets 4–8 were experimentally measured data and have been described in detail previously [41], [43]. Briefly, Phantom Set 4 consisted of 51 phantoms with varying SO_2_ levels but with a fixed [THb] (14.8 µM), and μ_s_' level (12.6 cm^−1^). Phantom Set 5 consisted of two subsets of phantoms with a low scattering level (μ_s_' = 13.5 cm^−1^) and high scattering level (μ_s_' = 22.52 cm^−1^). Each set in Phantom Set 5 consisted of 4 phantoms. Each phantom in the low scattering level was paired with a phantom in the high scattering level and the [THb] value of each paired phantom was the same. The standard deviation of the reflectance for each wavelength-pair in each paired phantoms were computed. Phantom Set 6 consisted of 13 phantoms with increasing [THb] from 5.86–35.15 µM. The averaged μ_s_' levels decreased for each phantom from 23.63 to 17.30 cm^−1^. A second instrument was used to measure the phantoms for Phantom Set 7 and Set 8 to validate the instrument independence of the ratiometric analysis. Phantom Set 7 was similar to Phantom Set 5 in that it contained two sets of 4 phantoms with low and high scattering levels (μ_s_' = 13.5 cm^−1^ and 22.89 cm^−1^ respectively) and paired phantoms from each level contained the same [THb]. The standard deviation of the reflectance for each wavelength-pair in each paired phantoms were also computed. Phantom Set 8 consisted of 16 phantoms with increasing [THb] from 5–50 µM. The μ_s_' level of each phantom was lower than the previous phantom, ranging from 28.56 to 17.02 cm^−1^, due to serial dilutions of the phantom solution. The combination of all of these experimental tissue phantoms measured serves to determine the best ratios to estimate [THb] and SO_2_ for a wide range of optical properties measured by different instruments.

**Table 2:** Optical properties for simulated and experimental tissue-mimicking phantoms.

Set	Type	Instrument	Bandpass (nm)	SO_2_	[THb] (μM)	<μ_s_'> (cm^−1^)
1	Simulation	-	2	0–1	5–50	8.9–26.6
2	Simulation	-	5	0–1	5–50	8.9–26.6
3	Simulation	-	10	0–1	5–50	8.9–26.6
4	Experiment	A	2	0–1	14.8	12.6
5	Experiment	A	2	1	6.4–14.3	13–21.7
6	Experiment	A	2	1	5.9–35.2	17.3–23.6
7	Experiment	B	3.5	1	7.3–16.2	14.3–21.9
8	Experiment	B	3.5	1	5.0–50.0	13.0–21.9

The ratiometric analysis was first tested on the simulated reflectance. Linear analytical equations for [THb] ratios and the non-linear logistic equations for SO_2_ ratios were generated from Phantom Sets 1–3. The extracted values of [THb] using the ratiometric analysis were compared to the true values for each diffuse reflectance spectrum and the absolute errors between the predicted and true values were calculated. Next, the sensitivity of each [THb] ratio to scattering was computed using the standard deviation of the reflectance ratio at each [THb].

The calculation of [THb] using the ratiometric analysis was also validated in Phantom Sets 4–8. Since every reflectance spectrum simulated by the MC model needs to be scaled by a calibrating phantom, the choice of the calibrating phantom can introduce systematic errors. To account for these effects on the extracted [THb], 3 different phantoms in Phantom Set 4, Set 6 and Set 8 and 2 different phantoms in Sets 5 and 7 were selected as the calibrating phantoms. The SO_2_, [THb] and μ_s_' of the calibrating phantoms are summarized in Table 2. Each time a calibrating phantom was selected, a new master set of reflectance was generated with the scalable MC model, and new coefficients for analytical equations were generated from these phantom sets. The generated analytical equations were used to extract the [THb] or SO_2_ values in the same experimental phantom sets from which the calibrating phantoms were selected. This ensured that the systematic errors or titration errors in one experimental phantom study were restricted to the same experimental phantom study and were not carried to another experimental phantom study. The probe geometries and bandpasses for the simulated master sets were matched to the experimental system. The ratiometrically extracted [THb] were compared to the MC extracted [THb] of the experimental phantoms for each phantom in Sets 4–8 to compute the absolute errors. The ratio spreads of the ten possible isosbestic wavelength pairs were computed from the paired phantoms in Set 5 and Set 7. The best ratio for [THb] was determined from the error and ratio spread rankings both with the simulated data and with the experimental data.

The ratiometric analysis for SO_2_ was validated in Phantom Set 4, which consisted of phantoms with varying SO_2_ levels. For each experimental phantom in this set, [THb] was first computed using the best isosbestic wavelength-pair using the ratiometric analysis. This extracted [THb] was then used to select the corresponding Hill curve coefficients for a given SO_2_ wavelength-pair. The reflectance ratio of each SO_2_ wavelength-pair was first computed and then converted to a SO_2_ value with the corresponding Hill curve coefficients. The ratiometrically extracted SO_2_ values were compared against the SO_2_ values measured with a pO_2_ electrode, as previously described [43]. To evaluate the sensitivity of each SO_2_ ratio to scattering, the reflectance ratios of each SO_2_ wavelength-pair were first computed in every phantom of Phantom Sets 5 and Set 7. The standard deviations were then computed from each paired reflectance ratios for each SO_2_ wavelength-pair since only the scattering was different within each paired phantom. The derived standard deviations from every paired phantom in Phantom Set 5 and Set 7 were averaged for each SO_2_ wavelength-pair.

### Instrumentation used in phantoms and clinical studies

**Figure 3:**
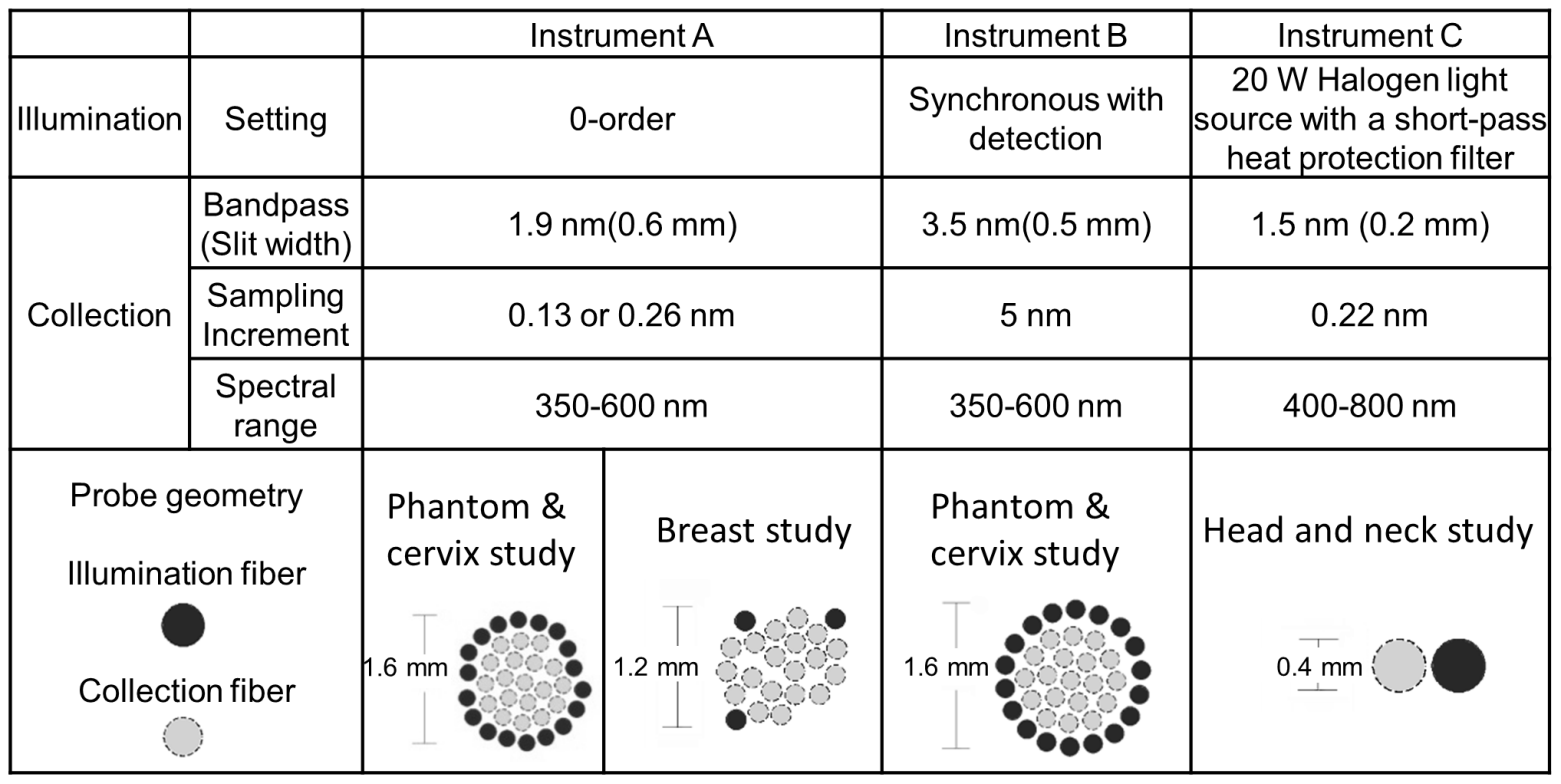
Illumination and collection parameters of the instruments used in experimental phantoms and clinical studies.

Three instruments were used to validate the ratiometric analysis in this manuscript. Instrument A was used in the experimental phantom studies (Set 4–6) and in an *in vivo* cervical study [41], [43] [44]. Instrument B was also used in the experimental phantom studies (Set 7–8), and also in the *in vivo* cervical study [44] and in an *in vivo* breast cancer study [45]. Instrument C was used for an *in vivo* head and neck cancer study. The details of Instrument A, B and C and the probe geometries have been previously described [44]–[47]. Briefly, Instrument A consisted of a 450 W xenon (Xe) arc lamp (JY Horiba, Edison NJ), double-excitation monochromators (Gemini 180, JY Horiba, Edison, NJ), and a Peltier-cooled open-electrode charge-coupled device (CCD) (Symphony, JY Horiba, Edison, NJ) [45] [43] [44]. Instrument B was a fiber-coupled spectrophotometer (SkinSkan, JY Horiba, Edison, NJ), which consisted of a 150 W Xe arc lamp, a double-grating excitation monochromator, an emission monochromator, and an extended red photomultiplier tube (PMT) [44] [43]. Instrument C was a portable system, which consisted of a 20 W halogen lamp (HL2000HP; Ocean Optics, Dunedin, FL), heat filter (KG3, Schott, Duryea, PA), and an USB spectrometer (USB4000, Ocean Optics, Dunedin, FL) [47]. Illumination and collection for all instruments were achieved by coupling to fiber optic probes. The instrument parameters are listed in Figure 3.

### Testing the ratiometric analysis with various scattering powers

The power law (μ_ s_' = *a*·λ^−*b*^) was used to model the reduced scattering coefficients where *a* determines the overall magnitude of scattering, λ is wavelength, and *b* is the scattering power. A new set of 1500 reflectance spectra (10 [THb] levels, 5 SO_2_ levels, and 10 different scattering powers with the scattering values equal to 2, 6, or 10 cm^−1^ at 600 nm) were simulated with the forward Monte Carlo model using the scattering coefficient generated from the power law. The scattering power was varied from 0.2 to 2 with steps of 0.2. The [THb] were range from 5 to 50 µM in steps of 5. The SO_2_ levels were range from 0 to 1 with increment of 0.25. Table 3 summarizes the optical properties used for testing the ratiometric analysis with various scattering powers. The [THb] and the SO_2_ were extracted with the ratiometric analysis for the best ratios determined in Section 3.1. The absolute [THb] and SO_2_ errors were computed. In addition, the scattering powers of the clinical data in this manuscript were computed by fitting the Monte Carlo-extracted wavelength-dependent scattering coefficients to the scatter power model.

**Table 3:** Optical properties used for testing the ratiometric analysis with various scattering powers.

			Avg. μ_s_' (350∼600 nm)		
[THb] (μM)	SO_2_	Scattering Power	μ_s_' = 2 (cm^−1^) at 600 nm	μ_s_' = 6 (cm^−1^) at 600 nm	μ_s_' = 10 (cm^−1^) at 600 nm
5–50	0–1	0.2	2.05	6.17	10.28
5–50	0–1	0.4	2.11	6.34	10.57
5–50	0–1	0.6	2.17	6.52	10.87
5–50	0–1	0.8	2.24	6.71	11.18
5–50	0–1	1	2.30	6.91	11.51
5–50	0–1	1.2	2.37	7.11	11.84
5–50	0–1	1.4	2.43	7.32	12.20
5–50	0–1	1.6	2.51	7.54	12.56
5–50	0–1	1.8	2.59	7.77	12.94
5–50	0–1	2	2.67	8.00	13.34

### Comparison of the speed of the MC and ratiometric analyses

To compare the computational performance of the ratiometric analysis and the full spectral MC analysis for extraction of [THb] and SO_2_, 100 diffuse reflectance spectra with randomly selected [THb] and SO_2_ values were simulated with the forward MC model. Random white noise was also added to each simulated reflectance spectrum before the fitting process. The amplitude of the generated random noise was limited to two percent of the difference between the simulated maximum and the minimum values of each reflectance spectrum. The noise level was determined from our previous study in which the worst SNR of instrument A is 44.58 dB. This means the amplitude of the noise is about two percent of the amplitude of the signal. These spectra were then analyzed using both the inverse full spectral MC analysis and the ratiometric analysis. The ratiometric analyses on these samples used the best ratios, which are described in the subsequent sections of this manuscript, for [THb] and SO_2_. The extracted [THb] and SO_2_ values for the full spectral MC analysis and the ratiometric analysis were compared to the expected (input) values and absolute errors were computed. The data processing time for both analyses were also compared.

### Clinical validation

To test the robustness of the ratiometric analysis in *in vivo* clinical settings, we applied the ratiometric analysis in three separate studies conducted on three different tissue sites. These clinical studies used diffuse reflectance spectroscopy to differentiate normal versus malignant or precancerous tissues *in vivo* in the cervix [44], in the breast [45], and in the head and neck [34]. The samples from these studies represent different optical absorption scenarios. Head and neck [34] and breast tissues have relatively high [THb] while the cervix has [THb] values at the lower end of the spectrum [44]. The ranges of [THb] from our previous results were 2.6–208.9 µM, 0.79–63.7 µM and 0.99–44.06 µM, for the head and neck, breast, and cervical tissues, respectively. In addition, breast tissue contains not only [THb] but also β-carotene as an additional absorber [45]. Data previously collected for the clinical studies and analyzed with the scalable full spectral MC analysis were used to evaluate the ratiometric analysis. The study designs and the protocols of these *in vivo* studies are described previously [44], [45]. All clinical studies in this manuscript were reviewed and approved by the Duke University School of Medicine Institutional Review Board. Written informed consents were obtained from each patient in these *in vivo* studies. The averaged diffuse reflectance spectrum for each site from each study was analyzed with both the inverse full spectral MC analysis and the ratiometric analysis. Pearson correlation coefficients between the full spectral MC and ratiometric analysis extracted [THb] and SO_2_ values were calculated for each clinical study.

In the cervical study, patients referred from the Duke University Medical Center (DUMC) Colposcopy Clinic after abnormal Papanicolaou tests were recruited. A fiber optic probe was used to deliver and collect the diffuse reflectance (350–600 nm) from one to three visually abnormal sites immediately after colposcopic examination of the cervix with the application of 5% acetic acid. This was followed by an optical measurement on a coloposcopically normal site from the same patient. Optical measurements of colposcopically normal and abnormal sites were taken prior to biopsy to avoid confounding absorption due to superficial bleeding. Diffuse reflectance from 76 sites in 38 patients were normalized by a reflectance standard and interpolated prior to calculating the reflectance ratios. Reduced scattering coefficients, [THb] and SO_2_ were also extracted from the same data using the inverse full spectral MC analysis [42].

For the head and neck cancer *in vivo* study, 42 enrolled patients had undergone panendoscopy with biopsy at Durham Veterans Administration Medical Center or DUMC. After the consented patient was under general anesthesia, the optical probe was placed on at least two sites: a clinically suspicious site and a distant normal site with normal mucosa appearance whose location was contralaterally matched to the suspicious site. At least 5 diffuse reflectance spectra were measured for each site. The biopsies were obtained immediately after the probe was removed from the measured clinical suspicious sites. All measurements were calibrated to the reflectance standard measured on the day of the surgery. In this head and neck study, the utility of the physiological and morphological endpoints obtained via the quantitative diffuse reflectance spectroscopy technique was investigated for the classification of head and neck squamous cell carcinoma at the time of staging panendoscopy. Malignant and non-malignant tissues were initially stratified by diagnosis and further classified by anatomical and morphological groupings to determine the most effective approach to discriminate squamous cell carcinoma (SCC) from its benign counterparts.

In the breast cancer study, thirty-five patients undergoing either a modified radical mastectomy or partial mastectomy for invasive and noninvasive breast malignancies were recruited at DUMC. The surgeon first located the lesion under ultrasound guidance; then, either a 10-gauge or 14-gauge biopsy needle coaxial cannula was guided through a small incision in the skin into the region of interest. A diffuse reflectance measurement (350–600 nm) was collected at a distance of 2 mm past the cannula with a fiber-optic probe after the removal of the needle and residual blood in the field. The optical probe was then retracted, and a biopsy needle was inserted through the cannula and a biopsy sample was removed. This resulted in the removal of a typically 20-mm-long cylinder of tissue, the proximal end of which corresponded to the volume optically measured by the probe. Tissue reflectance spectra from biopsies were normalized by the diffuse reflectance measured from an integrating sphere (Labsphere. Inc. North Sutton. NH) at the same day of the surgery for each patient. Biopsy samples were further processed through standard histologic procedures for pathological information.

### Comparing the classification performances of the full spectral MC and the ratiometric analyses

To compare the classification performances of the full spectral MC and ratiometric analyses, we compared the area under the receiver operating curves (AUC) calculated from the logistic regression models built based on the optical biomarkers extracted from the two analyses. We believe the AUC is more representative for the classification performance since the AUC is generated from various cut-off criteria. Since the full spectral MC model is able to extract optical biomarkers rather than just [THb] and SO_2_, we also included μ_s_' extracted with the for the full spectral MC model to build the logistic regression model for the cervix, breast and the head and neck groups. Beta-carotene concentrations extracted with the full spectral MC model were also included to build the logistic regression model for the breast group. The extracted [THb], μ_s_' and the beta-carotene concentrations were log transformed before building the logistic regression model. The *p* values were computed based on the method published by DeLong et al [48]. for comparing the ROC curves. All logistic regression models and the *p* values are computed with the SAS software (SAS Institute Inc., Cary, NC, USA)..

## Results

### The best ratio for estimating [THb] and SO_2_ from simulated and experimental phantoms

**Figure 4:**
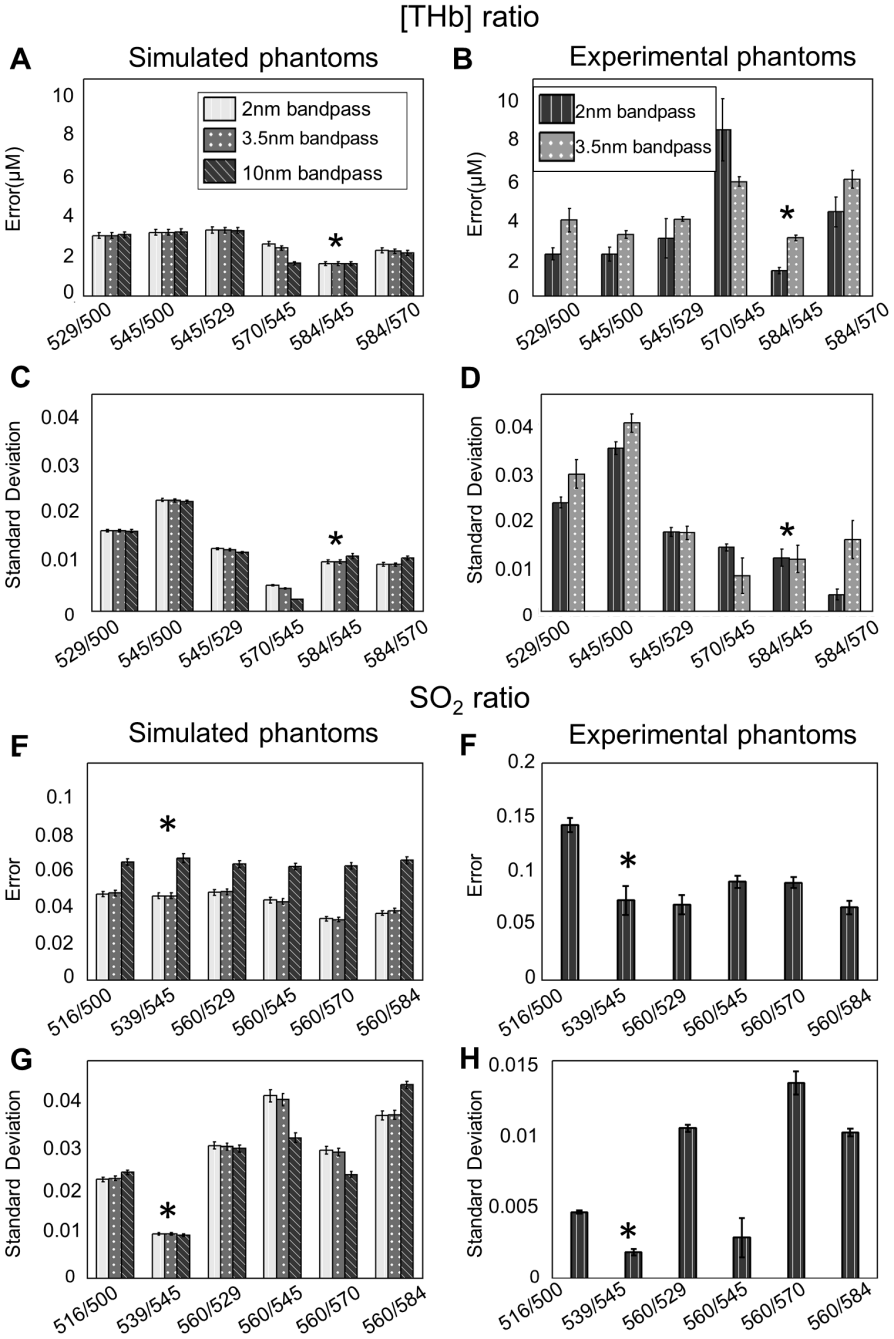
Results for the simulated phantoms and the experimental phantoms. Errors and ratio standard deviation of [THb] ratios and SO_2_ ratios from simulated phantoms and experimental phantoms. The top 6 ratios as defined by the lowest errors are shown. (A–B) Errors of the top 6 [THb] ratios in simulated data and experimental data. 584/545 has the lowest errors in both simulated phantom data and experimental phantom data. (C–D) Standard deviations of the top 6 [THb] ratios in the simulated data and the experimental data. 570/545, 584/545, and 584/570 have low standard deviation in both data sets. (E–F) Errors of the top 6 SO_2_ ratios in the simulated and experimental data. The errors are comparable for these ratios except for 516/500, which has higher errors in the experimental data. (G–H) Standard deviations of the top 6 SO_2_ ratios in the simulated data and the experimental data. 539/545 has the lowest standard deviation in both data sets. The best ratios for extracting [THb] or SO_2_ are marked with asterisk (*).

The accuracy of the 10 isosbestic wavelength-pairs to extract [THb] was evaluated in both simulated and experimental phantoms. Errors in extracted [THb] for each ratio were calculated. Next, the standard deviation of each ratio for changes in tissue scattering and SO_2_ was computed using only the simulated data. The 10 ratios were then ranked using both the standard deviations and the errors. The best ratio should be able to accurately extract [THb] with low sensitivity to both tissue scattering and SO_2_. A total of 25 wavelength-pairs were available for the calculation of SO_2_. The accuracy of these wavelength-pairs to determine SO_2_ was also ranked using an identical metric as was used for [THb]. Again, the best ratio should be able to accurately extract SO_2_ with low sensitivity to tissue scattering. Figures 4A and 4B show 6 ratios with the lowest errors to extract [THb] in the simulated and experimental datasets, respectively. Figures 4C and 4D shows the standard deviation of the [THb] ratios for various SO_2_ and scattering levels in the simulated and experimental data, respectively. Figures 4E-4H show similar data for SO_2_. For [THb] ratios, 584/545 has the lowest average errors for each band pass in both simulated and experimental phantoms. The standard deviation of 584/545 was the third lowest for each band pass in simulated data and the second lowest for each band pass in experimental phantoms. This means that 584/545 can extract [THb] with relatively small errors, and it is relatively insensitive to the scattering or SO_2_. The average errors are comparable in both simulations and in experimental phantoms for the top 6 SO_2_ ratios with the exception of 516/500, which has higher errors in the experimental phantom. 539/545 has the lowest average ratio and standard deviation in both simulation and experimental phantoms. Thus, 584/545 and 539/545 were chosen as [THb] and SO_2_ ratios for further testing.

### Testing the ratiometric analysis with varying scattering powers

**Figure 5:**
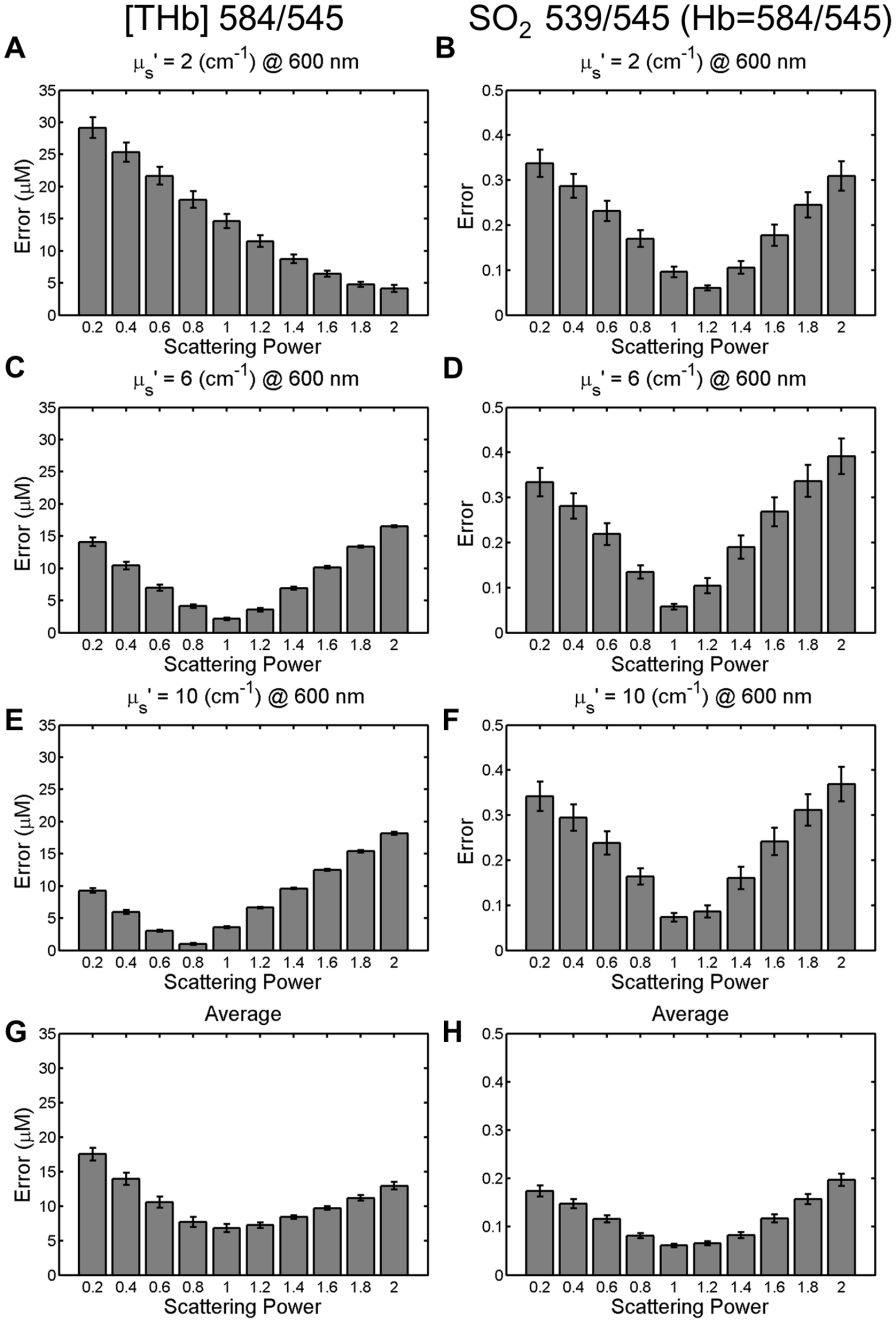
Average absolute error for best [THb] and SO_2_ ratios tested with various scattering powers. (A), (C), (E) Absolute errors for extracting the [THb] of the simulated reflectance spectra with 584/545 when the scattering power varied from 0.2 to 2 for different scattering levels. (B), (D), (F) Absolute errors for extracting the SO2 of the simulated reflectance spectra with 539/545 when the scattering power varied from 0.2 to 2 for different scattering levels. (G) Averaged errors from (A), (C) and (E). (H) Average errors from (B), (D) and (F). Error bars represent the standard errors.

Figure 5 shows the absolute errors of the extracted [THb] and SO_2_ for the best [THb] and SO_2_ ratios when using the scatter power model. The accuracies for extracting [THb] and SO_2_ varied with scattering power. In our data, the average and the standard deviation of the scattering power for head and neck, cervix and breast tissues are 0.62±0.12, 0.55±0.27 and 0.50±0.16 respectively.

### Comparison of the speed of the MC and the ratiometric analyses

**Figure 6:**
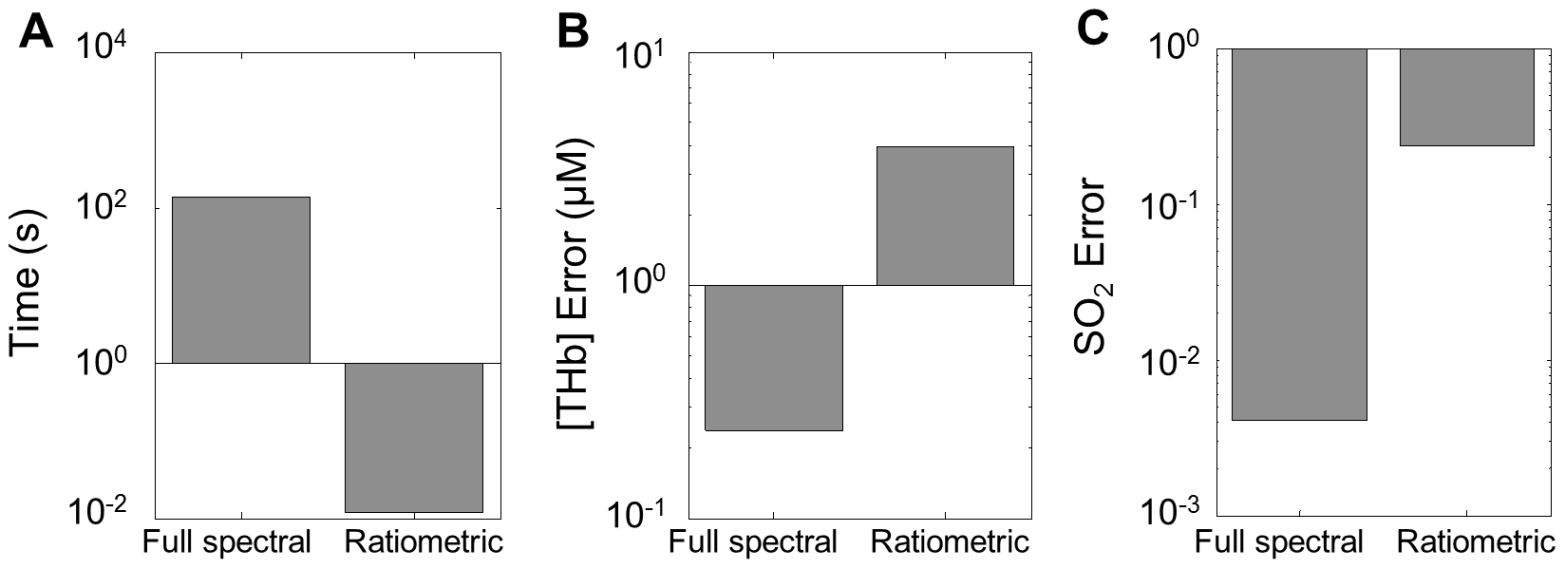
Comparison between the scalable inverse MC analysis and the ratiometric analysis. (A) Elapsed time of extracting 100 MC simulated phantoms for the scalable inverse MC model and the ratiometric analysis. (B) Absolute [THb] error and (C) SO_2_ errors for MC and ratiometric analysis.

Figures 6A–C show the comparison of the computational time, the mean error in [THb] extraction, and the mean error in SO_2_ extraction using the scalable full spectral MC analysis and the ratiometric analysis. These data were generated using 100 simulated diffuse reflectance spectra. [THb] was extracted using the ratiometric analysis with the ratio computed between 584 nm and 545 nm. The extracted [THb] value from the ratiometric analysis was then used to determine the look-up coefficients to calculate the SO_2_ using the 539 nm/545 nm ratio. As shown in Figure 6A, the ratiometric analysis is over 4000 times more computationally efficient compared to the full spectral MC analysis [42]. Figures 6B and 6C show the mean error for [THb] extraction and SO_2_ extraction using the full spectral MC analysis and the ratiometric analysis. The mean errors were 0.24 µM and 3.94 µM for [THb] extraction, while the errors were 0.004 and 0.23 for SO_2_ values for the MC analysis and the ratiometric analysis, respectively.

### Comparison of MC and ratiometric analysis

Correlation coefficients were computed between the optical endpoints extracted using both analyses for each tissue group in each of the three clinical studies. Table 4 summarizes the Pearson correlation coefficients between the full spectral MC analysis and the ratiometric analysis for [THb] and SO_2_ of each tissue group in the cervical pre-cancer, head and neck squamous cell carcinoma, and breast cancer studies. The normal samples in the breast cancer study were further classified into the benign and adipose group, depending on the adipose percentage of the normal sample [45]. The overall correlation coefficient for each study was also computed when all samples in each study were used.

**Table 4:** Correlation coefficients and *p*-values between the ratiometric analysis and the Monte Carlo analysis.

		[THb]		SO_2_	
Study		r	*p*	r	*p*
Cervix	All Tissues	0.69	<0.01	0.43	<0.01
	Normal	0.66	<0.01	0.34	0.02
	CIN1	0.62	0.01	0.5	0.05
	CIN2+	0.76	<0.01	0.46	0.13
Head and neck	All Tissues	0.92	<0.01	0.87	<0.01
	Glottic	0.97	<0.01	0.91	<0.01
	Lymphoid	0.72	<0.01	0.85	<0.01
	Mucosal	0.92	<0.01	0.87	<0.01
Breast	All Tissues	0.77	<0.01	0.71	<0.01
	Tumor	0.85	<0.01	0.63	<0.01
	Benign	0.71	<0.01	0.56	<0.01
	Adipose	0.82	<0.01	0.48	<0.01

### Accuracy of algorithm in cervical tissues with low tissue vascularity

**Figure 7:**
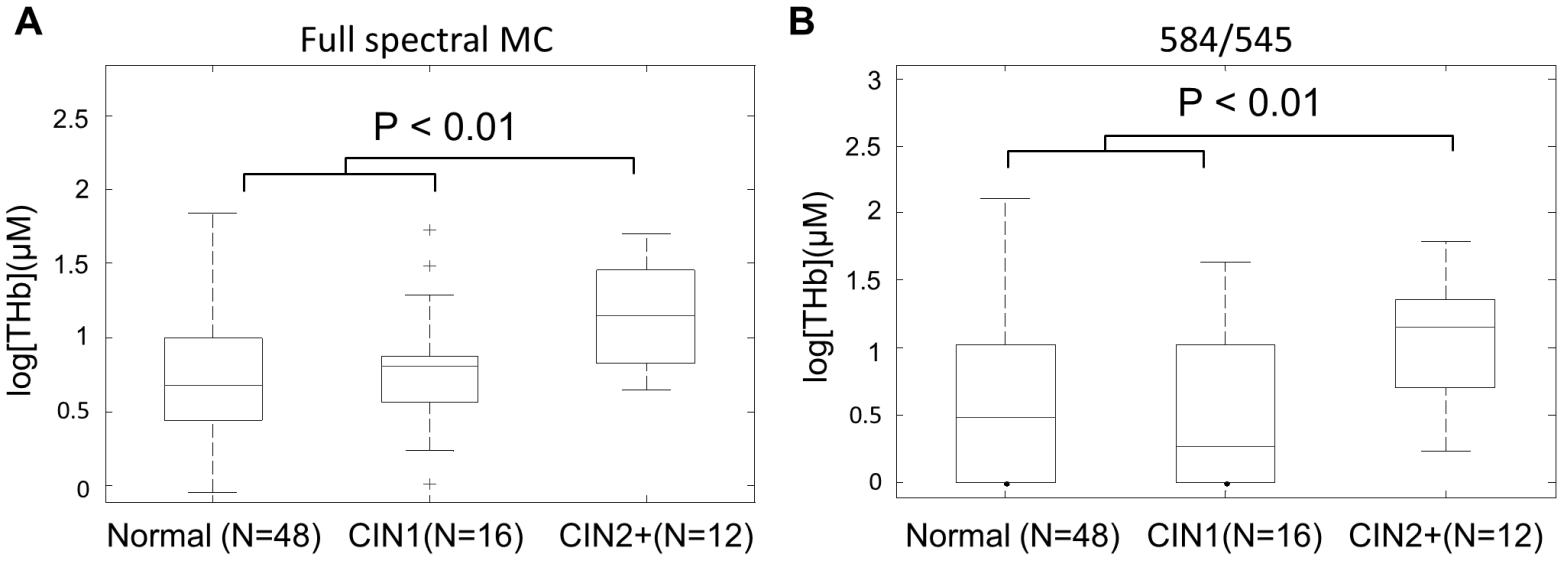
Results for the *in vivo* cervix study. Boxplots for (A) full spectral Monte Carlo extracted and (B) ratiometrically (584/545) extracted [THb]. Unpaired two-sided student *t*-tests were performed between normal, CIN1, and CIN2+. Significant *p*-values are shown.

[THb] was extracted using the inverse full spectral MC analysis from a total of 76 samples from 38 patients, as published previously. The samples were classified as normal, low-grade cervical intraepithelial neoplasia (CIN 1) and high-grade cervical intraepithelial neoplasia (CIN 2+). Figure 7A shows boxplots for the full spectral MC extracted [THb] for the three tissue groups. Figure 7B shows boxplots for [THb] extracted using the ratiometric analysis for the three tissue groups. To compare with the previously published results extracted by the full spectral MC analysis, a log transformation was applied to the ratiometrically-extracted [THb]. [THb] determined using both analyses was statistically higher in CIN2+ tissues (*p*<0.01) compared to normal and CIN1 samples. No statistical differences were found when comparing the SO_2_ of different tissue groups with the full spectral MC analysis or the ratiometric analysis. The *p*-values were derived from the unpaired two-sided student *t*-tests for consistency with the previously published data [44].

### Accuracy of algorithm in head and neck tissues with high vascularity

**Figure 8:**
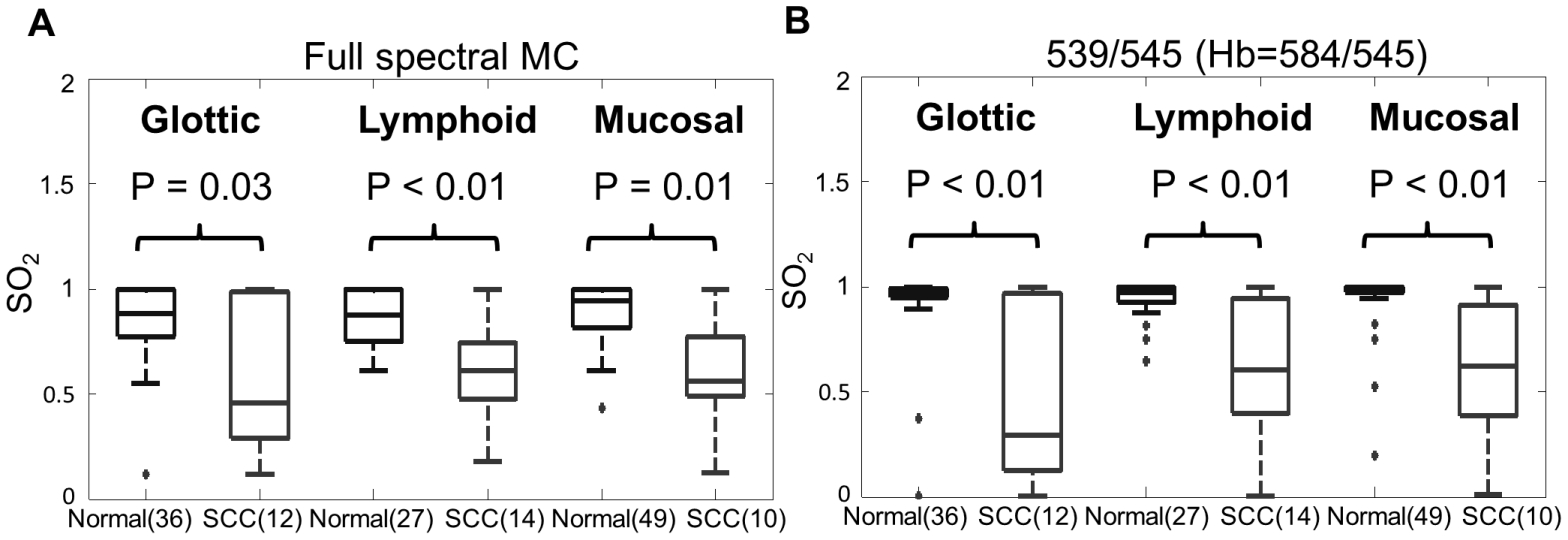
Results for the *in vivo* head and neck study. Boxplots for SO_2_ at malignant and normal sites extracted with the (A) full spectral Monte Carlo analysis and the (B) ratiometric analysis. Wilcoxon rank-sum tests were performed between normal and SCC sites for each tissue type. Significant *p*-values from are shown. (SCC: squamous cell carcinoma).

Figure 8 shows boxplots for SO_2_ values extracted with full spectral MC analysis and the ratiometric analysis, across all measured tumor and normal sites in head and neck squamous cell carcinoma patients. The samples were separated into 3 groups (glottic, lymphoid and mucosal) based on morphological location of each measurement site. Wilcoxon rank-sum tests were used to establish differences between the extracted SO_2_ values in the normal and SCC sites, for each tissue group. The extracted SO_2_ was significantly different between SCC and normal samples for the glottic, lymphoid and mucosal tissue groups when extracted using both the full spectral MC analysis (*p* = 0.03, *p*<0.01 and *p* = 0.01 respectively) and the ratiometric analysis. SO_2_ values extracted using the ratiometric analysis (*p*<0.01 for the 3 groups) showed similar differences between the SCC and normal samples for these tissue groups.

### Accuracy of algorithm in breast tissues with multiple absorbers

**Figure 9:**
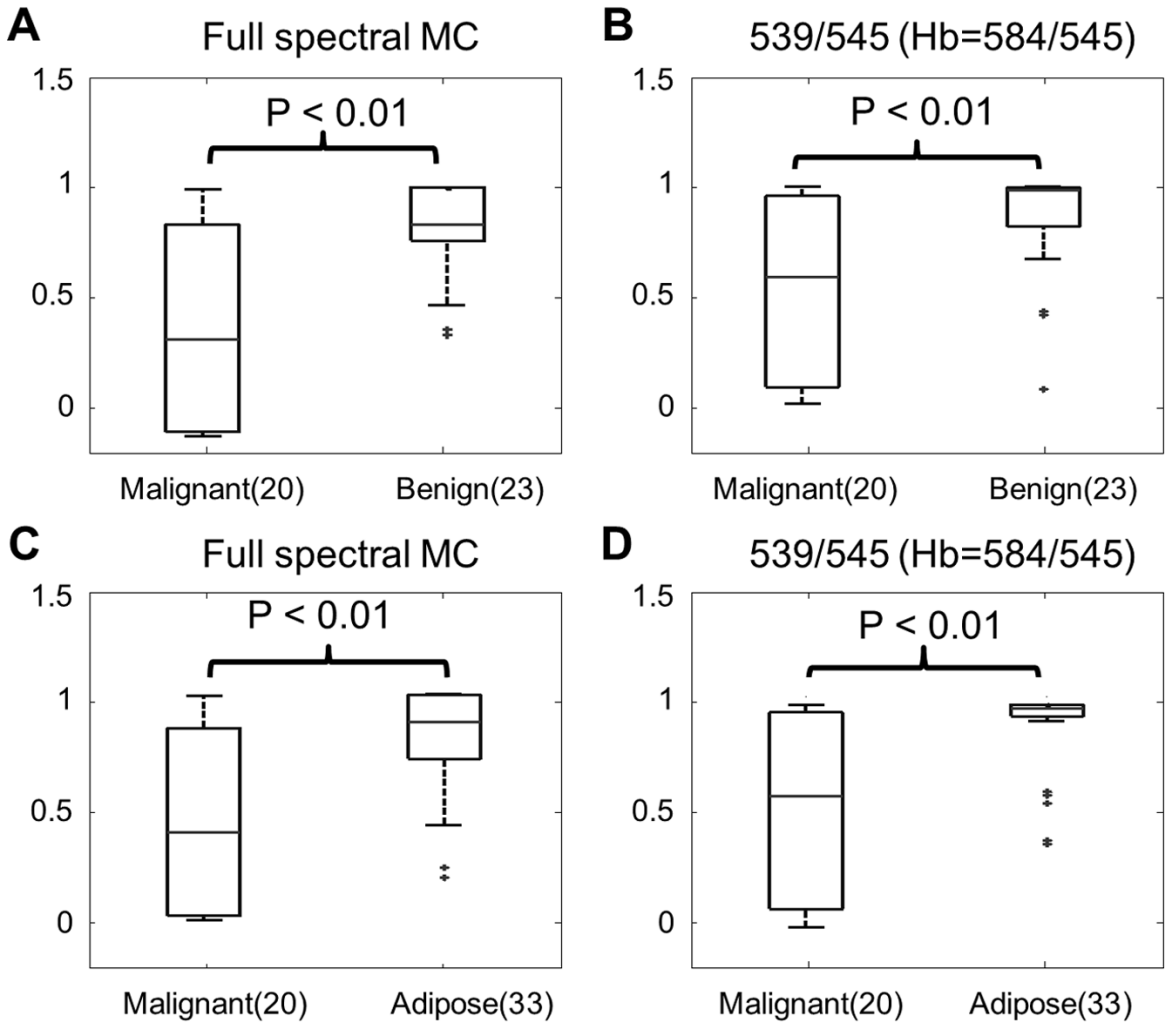
Results for the *in vivo* breast study. Boxplots for SO_2_ of the malignant and benign samples extracted with the (A) full spectral Monte Carlo analysis and (B) the ratiometric analysis. Boxplots for SO_2_ of the tumor and adipose samples extracted with the (C) full spectral Monte Carlo analysis and (D) the ratiometric analysis. Ratiometric [THb] was estimated with 584/545, and SO_2_ was estimated with 539/545. Wilcoxon rank-sum tests were performed for each group, and the significant *p*-values are shown.

Figure 9 shows a boxplot for the inverse full spectral MC or the ratiometrically extracted SO_2_ of malignant and normal breast tissues. The normal samples were reclassified into a benign group if the fat content of the tissue biopsy was less than 50% or into the adipose group if the fat content in the biopsy was greater than 50% as described previously [45]. Figure 9A shows boxplots for the full spectral MC extracted SO_2_ of the tumor and benign tissues whereas Figure 9B shows boxplots for the ratiometrically extracted SO_2_ of tumor and benign tissue from the *in vivo* breast study. Figures 9C and 9D also show boxplots for the SO_2_ of the tumor and adipose tissues extracted with both analyses. Wilcoxon rank-sum tests were performed to test the statistical significance of the extracted SO_2_ between the tumor samples and normal (both benign and adipose) tissues for both full spectral MC analysis and ratiometric analysis. The extracted SO_2_ of the normal samples were significantly higher than the tumor samples (*p*<0.01) for both the ratiometric analysis and the full spectral MC analysis.

### Comparison the classification performances of the full spectral MC and the ratiometric analyses

**Figure 10:**
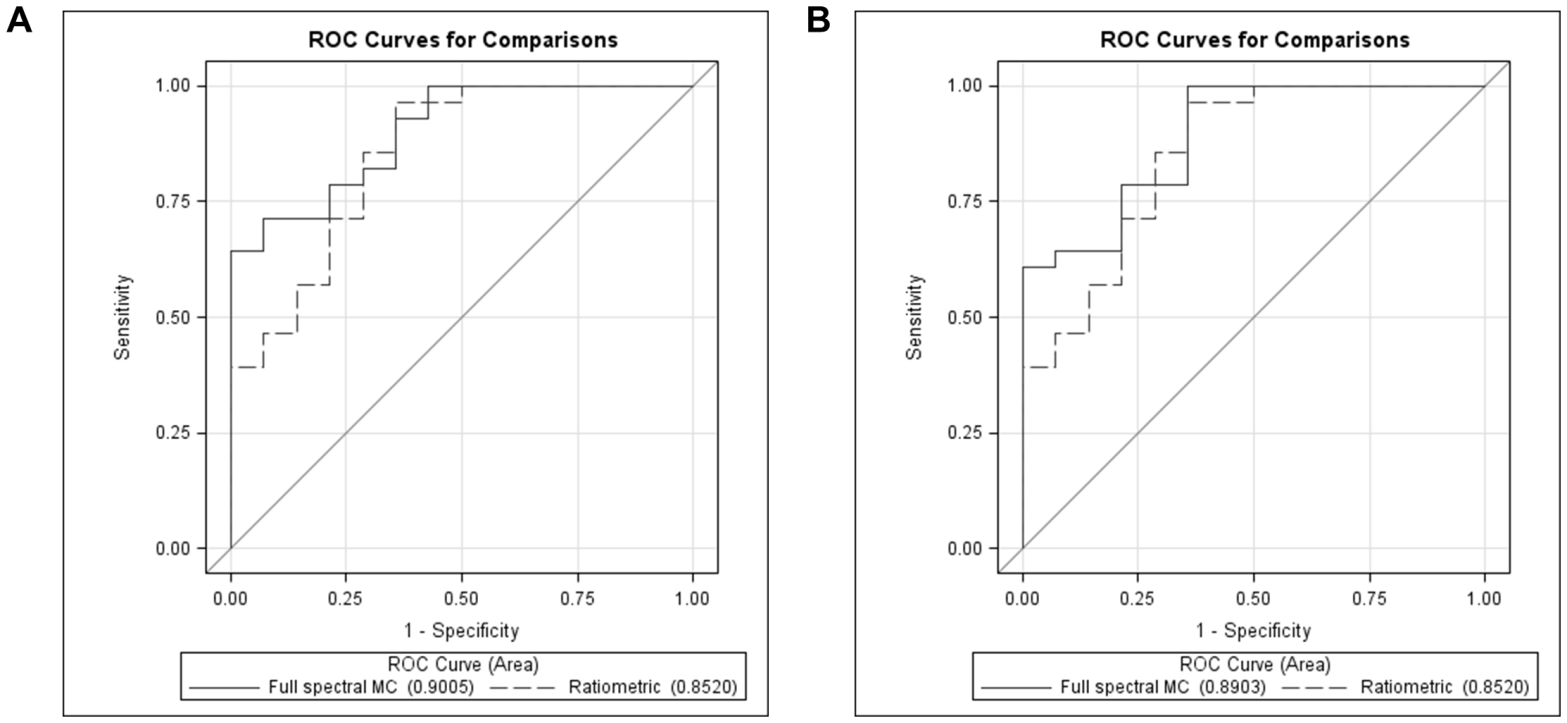
The area under the receiver operating curves computed from the logistic regression model built base on different optical biomarkers from the full spectral MC and the ratiometric analyses for lymphoid tissues. (A) Full spectral MC and the ratiometrically extracted SO_2_, log([THb]) were used for building the MC and the ratiometric logistic regression models respectively. (B) SO_2_, log([THb]), log(μ*_s_*') were used to build the logistic regression model for the full spectral MC analysis and the ratiometric ROC curve was built based on the SO_2_, log([THb]).

The combinations of the optical biomarkers used for building the logistic regression models and the area under the receiver operating curve (ROC) are summarized in Table 5. No significant *p* values were observed when comparing the AUC calculated between the two analyses. Representative ROC curves built based on the optical biomarkers extracted from the lymphoid tissues using the full spectral MC and the ratiometric analyses are also shown in Figure 10. The full spectral MC ROC curve in Figure 10A was built based on the SO_2_ and the log([THb]) and the full spectral MC ROC curve in Figure 10B was built based on SO_2_, log([THb]) and log(μ_s_'). Both ratiometric ROC curves in Figure 10A and 10B were built based on SO_2_ and log([THb]).

**Table 5:** The area under the receiver operating curves computed from the logistic regression model built base on different optical biomarkers from the full spectral MC and the ratiometric analyses.

Breast				
Full spectral MC optical biomarkers	AUC	Ratiometric optical biomarkers	AUC	*p* value
SO_2_, log([THb])	0.83	SO_2_, log([THb])	0.79	0.42
SO_2_, log([THb]), log(μ*_s_*')	0.85	SO_2_, log([THb])	0.79	0.26
SO_2_, log(THb), log(μ*_s_*'), log(β-carotene)	0.85	SO_2_, log([THb])	0.79	0.24
Cervix				
SO_2_, log([THb])	0.76	SO_2_, log([THb])	0.72	0.51
SO_2_, log([THb]), log(μ*_s_*')	0.77	SO_2_, log([THb])	0.72	0.54
Head and neck (Glottic)				
SO_2_, log([THb])	0.79	SO_2_, log([THb])	0.76	0.69
SO_2_, log([THb]), log(μ*_s_*')	0.83	SO_2_, log([THb])	0.76	0.48
Head and neck (Lymphoid)				
SO_2_, log([THb])	0.90	SO_2_, log([THb])	0.85	0.32
SO_2_, log([THb]), log(μ*_s_*')	0.89	SO_2_, log([THb])	0.85	0.40
Head and neck (Mucosal)				
SO_2_, log([THb])	0.81	SO_2_, log([THb])	0.86	0.13
SO_2_, log([THb]), log(μ*_s_*')	0.83	SO_2_, log([THb])	0.86	0.31

## Discussion

A simple and fast analysis for quantitative extraction of [THb] and SO_2_ of tissues is presented. The analysis used a look-up table that allows conversion of the ratio of the diffuse reflectance at two selected wavelengths into [THb] and SO_2_ values. This ratiometric analysis uses two isosbestic wavelengths for the calculation of [THb] and one isosbestic wavelength along with a wavelength where a local maximum difference in the extinction coefficients of deoxy- and oxy- hemoglobin exists for SO_2_. A total of 10 wavelength-pairs were tested for extraction of the [THb] while 25 wavelength-pairs were tested for SO_2_. The wavelength-pairs with the least dependence on tissue scattering were selected through rigorous tests on a total of 24805 spectra. The look-up tables used to translate the reflectance ratio into quantitative values were built for specific experimental probe-geometries and theoretically can be extended to any given source-detector configuration. Further, calibration using specific experimental phantoms ensured that the ratiometric analysis could directly be used on experimentally measured data. Once analytical equations for the ratiometric analysis were generated, extraction of [THb] and SO_2_ values from experimentally measured diffuse reflectance was over 4000 times faster than the scalable inverse full spectral MC analysis with minimal loss in accuracy. Even though the ratiometric analysis is not expected be as accurate as the inverse full spectral MC analysis, the ratiometric analysis achieves similar contrast between malignant and the benign tissues in three different organ sites for a wide range of tissue vascularity and for tissues with multiple absorbers.

A prominent hemoglobin absorption feature (Soret band) occurred around 410–420 nm in the visible spectrum. However, we omitted the absorption peaks of hemoglobin around the 410–420 nm since most silicon-based detectors have lower sensitivities in this region. In order to detect the hemoglobin absorption around 410–420 nm, higher power light sources or more sensitive detectors would be required. In order to leverage relatively low priced light sources, the wavelengths were chosen from 500 nm to 600 nm (visible spectrum) in this manuscript.

The purpose of the bandpass simulations was to understand if the best [THb] or the SO_2_ ratios would change for the different systems used. Our results show that 584/545 and 539/545 are the best ratios for the simulated results with three different bandpass values. Both 584/545 and 539/545 can extract [THb] or SO_2_ with low errors and both ratios have low sensitivity to scattering. Although different systems might have different bandpasses, the relative rankings of the [THb] ratios and SO_2_ ratios for error and the sensitivity to scattering remain the same. The clinical data has three different bandpasses. The band passes were 1.5 nm and 1.9 nm for the head and neck and breast data, respectively. The bandpasses were 1.9 nm or 3.5 nm for the cervical data. The extracted data with the ratiometric analysis show good agreement with the full spectral MC extracted values. In addition, the simulated [THb] results in Figures 4A and 4B are consistent except that 570/545 has higher errors in the experimental data. We also believe the 74415 (24805 spectra * 3 different bandpass values) MC-simulated spectra can account for a wide range of optical properties and thus, is more comprehensive than the experimental data.

**Figure 11:**
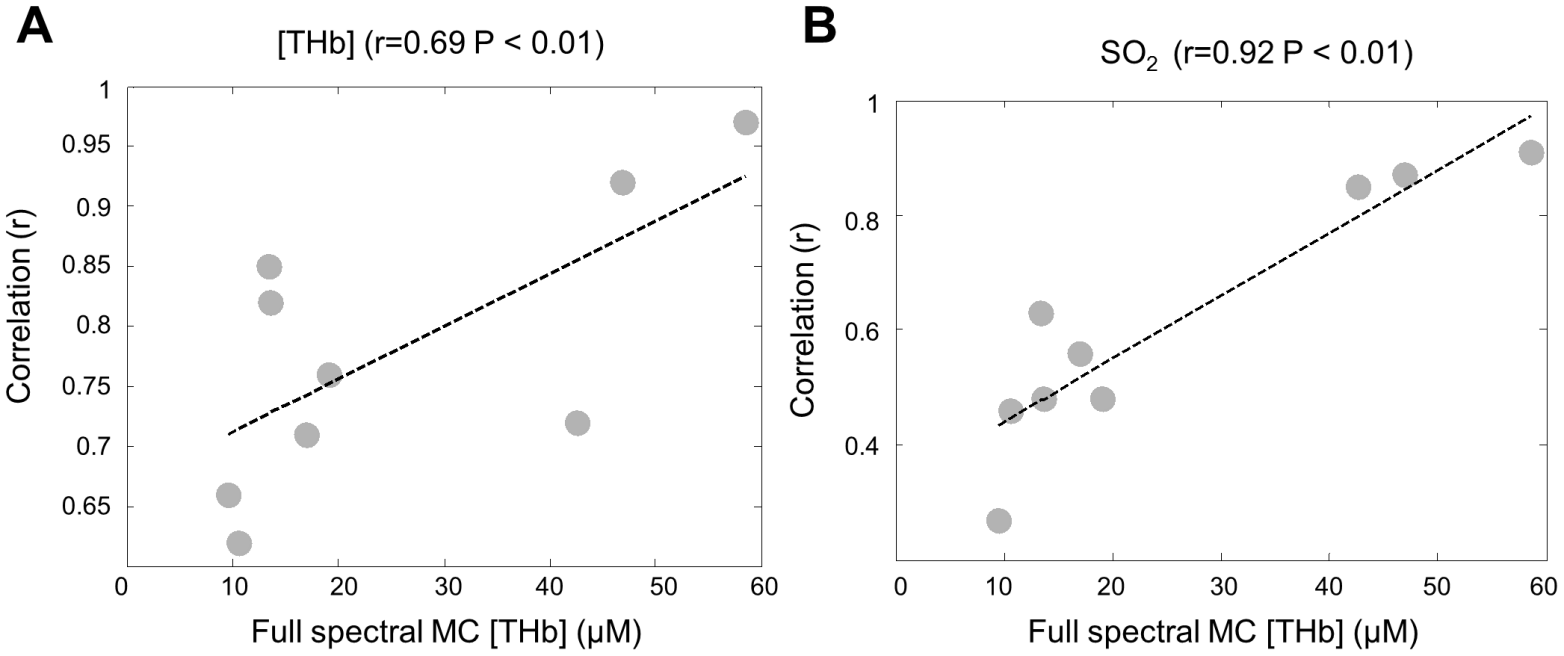
Correlation between Monte Carlo analysis and ratiometric analysis. (A) Correlation coefficients between the full spectral Monte Carlo and ratiometrically extracted [THb] as a function of the average full spectral Monte Carlo extracted [THb] for the 9 tissue groups in Table 4. (B) Correlation coefficients between the full spectral Monte Carlo and ratiometrically extracted SO_2_ as a function of the average full spectral Monte Carlo extracted [THb] for the 9 tissue groups in Table 4.

The sensitivities of the ratiometric analysis to the scattering power were tested since the scattering power is likely to change in the real tissues. As can be seen in Figure 5, the accuracies varied as the scattering power has changed. Although the ratiometric analysis is less accurate when the scattering power varies than when the scattering power is a constant, the contrast between malignant and non-malignant tissues in breast and head and neck or the contrast between the low-grade and the high-grade cervical tissues is still preserved. In addition, our analysis found a high degree of correlation in the extracted [THb] and SO_2_ values between the ratiometric analysis and the inverse full spectral MC analysis. These correlations were especially high for measurements in head and neck tissues. Correlations between the extracted [THb] and SO_2_ in cervical tissues were the lowest, relative to head and neck or breast tissues. These effects might be due to the fact that the [THb] was typically much higher in the head and neck and breast studies, relative to the cervical study (the averaged full spectral MC extracted [THb] for head and neck, breast and cervical tissues were, 57.8 µM, 14.2 µM and 5.9 µM respectively). In other words, correlations between the ratiometrically and the full spectral MC extracted [THb] or SO_2_ are positively correlated to the full spectral MC extracted [THb]. This can be seen in Figure 11, which shows the scatter plot for the average MC extracted [THb] for the 9 tissue groups in Table 4 versus the correlation coefficients between the full spectral MC extracted and ratiometrically-extracted [THb] and SO_2_. Because hemoglobin has very high extinction coefficients in the UV spectral-range relative to the visible, using wavelengths in the UV range could provide increased dynamic-range for sensing hemoglobin. This reasoning supports our previously published study [41], where the ratiometric technique for extraction of [THb] was superior for the 545/390, 452/390 and 529/390 ratios, relative to the 584/545 wavelength pair used here.

Although the ratiometric analysis was developed by assuming that hemoglobin was the primary absorber in tissue, the experimental measurements on human tissue can be influenced by absorbers other than hemoglobin [49] [50]. However, SO_2_ and [THb] are the hallmarks of carcinogenesis and represent the features of a growing tumor. This has been published on widely and is useful in diagnostics and therapeutics [51] [52] [53] [54] [55]. For example, neovascularization increases with the development of cancer, and tumor hypoxia occurs as tumors outstrip their blood supply. Thus, being able to measure these endpoints with an optical technology that is optimized for speed and cost will have applications in early detection, diagnostics and response to therapy. Although some tissues may have multiple absorbers in addition to Hb, the classification performances were not significantly affected when using only [THb] and SO_2_ as parameters (in cervix and head & neck only). Further, optical technologies have a significant potential to have an impact in global health. The ratiometric analysis still worked reasonably well in breast tissue, where beta-carotene is a known absorber in the wavelength range used. The presence of beta-carotene could be one reason why we obtained a slightly lower correlation coefficients between the ratiometric and full spectral MC analysis in the breast study, relative to the head and neck study. Overall, in all of the clinical studies, the [THb] extracted from the ratiometric analysis were better correlated to full spectral MC values, in comparison to the SO_2_ values. The effect of beta-carotene is more obvious in the SO_2_ estimation than in the [THb] estimation. This could possibly be due to the absorption of beta-carotene being 8.5 times lower in the 550–600 nm compared to 500–550 nm. However, despite the lower correlation for the SO_2_ estimation in the breast tissues, the ratiometric analysis is still able to preserve the contrast between the malignant and non-malignant breast tissues observed with the results using the full spectral MC analysis.

In this manuscript, we have specifically shown the potential utility of the ratiometric analysis for diffuse reflectance imaging. Since our ratiometric analysis only involves wavelengths at 539, 545 and 584 nm, this analysis can be incorporated into any system with the use of a simple white LED and appropriate bandpass filters. With appropriate optimization for wavelength and illumination and collection geometries, the ratiometric analysis might be applied to a variety of spectral imaging systems [56] [57] [58] [59]. For example, this analysis can be incorporated into previously developed fiber-less technology [59], where a Xenon lamp and light filters are used to illuminate the tissue at different wavelengths of light. The illumination light was delivered through free space with a quartz light delivery tube. A custom photodiode array is in contact with the tissue to directly measure diffuse reflectance from a large area of tissue. With proper modifications of this system and combined with the ratiometric analysis, real-time [THb] and SO_2_ imaging is possible.

## Conclusion

A rapid analytical ratiometric analysis for determining [THb] and SO_2_ in head and neck, cervical, and breast tissues was presented. This analysis is non-invasive, label-free, quantitative, and fast. The ratiometric analysis requires the diffuse reflectance only from three selected wavelengths to calculate both [THb] and SO_2_. Thus, the system design could be simple, portable, and potentially useful for global health applications. The fast computation speed allows near real-time [THb] and SO_2_ mapping of tissue. This will provide important physiological information for many clinical applications, from cancer screening to diagnostics to treatment.
